# Association between the procedure of tibiotalocalcaneal arthrodesis by hindfoot nailing and quality of life in Charcot’s joint

**DOI:** 10.1186/s13018-024-04787-9

**Published:** 2024-06-03

**Authors:** Shirvan Rastegar, Mehdi Teymouri, Jamal Sabaghi

**Affiliations:** Isfahan university of medical science/orthopedic department, Isfahan, Iran

**Keywords:** Charcot, Hindfoot, Nailing, Arthrodesis

## Abstract

**Introduction:**

Charcot arthropathy is a progressive disorder of the ankle and foot joints that can lead to foot deformity and instability. Surgical intervention is often necessary for deformity and ulcer management during the chronic phase. The device used for arthrodesis remains a challenge.

**Methods:**

This clinical trial study included diabetic patients aged 40 years or older with Charcot foot. Lateral approach with lateral malleolar osteotomy was used to access the ankle joints and remove the cartilage. A small incision was made on the plantar aspect of the foot to pass an appropriately sized intramedullary nail. Demographic information, medical history, surgical details and Clinical data were collected at 2-week and 1-year follow-ups using the Ankle-Hindfoot Scale (AOFAS) score and the EuroQol 5-Dimensional 5-Level (EQ-5D-5L) health utility score.

**Results:**

Twenty-six patients with a mean age of 63 ± 0.23 years were included in the study. The findings showed significant improvements in AOFAS questionnaire items related to pain score, length of the walk, walking surfaces, walking disorders, sagittal alignment, back leg alignment, sustainability, alignment and the total score (*P* value < 0.001). The EQ-5D-5L questionnaire also showed a significant improvement in the total score (*P* value = 0.002).

**Conclusion:**

This study provides evidence supporting the effectiveness of tibiotalocalcaneal arthrodesis by hindfoot nailing in diabetic patients with Charcot foot joints and demonstrated comparable and superior outcomes in terms of patient satisfaction and complication rate when compared to previous studies.

## Introduction

Charcot arthropathy is a progressive disorder of the ankle and foot joints that occurs in 0.1% to 7.5% of patients with diabetic foot ulcers and is considered one of the major challenges of orthopedic surgery [1]. Over 35% of Charcot feet progress their deformity and develop foot ulcers at median 2 year follow which decreases to 4% after Achilles lengthening [2]. Ulceration usually results in amputation (8.9%) because of abscess formation, gas gangrene and osteomyelitis [3].

The goal of managing Charcot foot is to maintain ambulation by preventing progression of deformity, ulceration & amputation. Patients have been treated initially in the past with offloading with walkers, knee walkers, braces and shoe modification. Surgery has usually been reserved for failure of conservative treatment. Initial or early treatment with Achilles lengthening has been recommended which can usually prevent progression of deformity, instability, ulceration and amputation [4].

Despite the many advantages of conservative treatment, more advanced cases require more extensive surgery to prevent progression of deformity to ulceration and amputation and to maintain ambulation.

The use of intramedullary rod fixation is biomechanically superior to plates, screws and external fixators since it allows the bone to share more of the load. IM rods are preferred in diabetics because of their loss of protective sensation causes loss of fixation to be more likely[5–8]. For these reasons we decided to evaluate the treatment of Charcot ankle ± subtalar deformity and instability with using Hindfoot tibiotalarcancaneal arthodesis nail.

## Materials and method

### Study design and location

The current clinical trial study was conducted after the approval of the Research Ethics Committees of the School of Medicine-Isfahan University of Medical Sciences (Ethical code: IR.MUI.MED.REC.1401.304) at the educational hospitals in Isfahan (January 2020–December 2021). Diabetic patients with Charcot's foot and hindfoot arthropathy who had inclusion criteria, age > 40 years, serum albumin > 3 gr/dl, serum vitamin D3 > 30 ng/ml, 4000 < WBC < 10,000 cell/micL, and HbA1c < 6.5% and who provided written consent were included in the study.

The exclusion criteria were patients with the foot or ankle ulcer, active foot or ankle infection, abscess or purulent discharge.

### Surgical procedure

To reduce human error, all patients were operated on by the same surgical team. After prepping and draping in the supine position, lateral approach with lateral malleolar osteotomy was used to access the ankle joints and remove the cartilage via subchondral bone curettage. A small incision was made on the plantar aspect of the foot to pass a guidewire from the calcaneus bone to the tibia bone via the talus, followed by reaming the tibia medulla and using an appropriately sized intramedullary nail. The wound was then washed and closed.

### Outcome measures

The primary data of the study were assessed using the Ankle-Hindfoot Scale (AOFAS) score and the EuroQol 5-Dimensional 5-Level (EQ-5D-5L) health utility score.

The AOFAS score is a clinical evaluation system that assesses a patient's pain and walking ability based on their perception and the physician's understanding, without considering radiographic results. In this questionnaire, scores between 100 and 90 are excellent, 89–80 are good, 79–70 are average, and below 70 are poor. The AOFAS questionnaire was validated in a study by Kitaika et al. [9]. The Persian form of the AOFAS was validated in a study by Sayyed-Hosseinian et al., with a Cronbach’s alpha coefficient of 0.696 [10].

The EQ-5D-5L is the most commonly used instrument for evaluating quality-adjusted life-years (QALYs). This instrument was first invented by Herdman et al. [11]. The descriptive system of the EQ-5D consists of five dimensions: mobility, self-care, usual activities, pain/discomfort and anxiety/depression. Each dimension is ranked on a five-point scale ranging from no problems [1] to extreme problems [5]. Patients indicate their health status by selecting the most appropriate statement for each dimension, resulting in a 5-digit code that describes their health status.

To calculate a summary index for an individual's EQ-5D health status, a value set is needed. In this study, we used a standard value set for the Iranian population based on the study of Ameri et al. [12].

### Statistical analysis

**Fig. 1:**
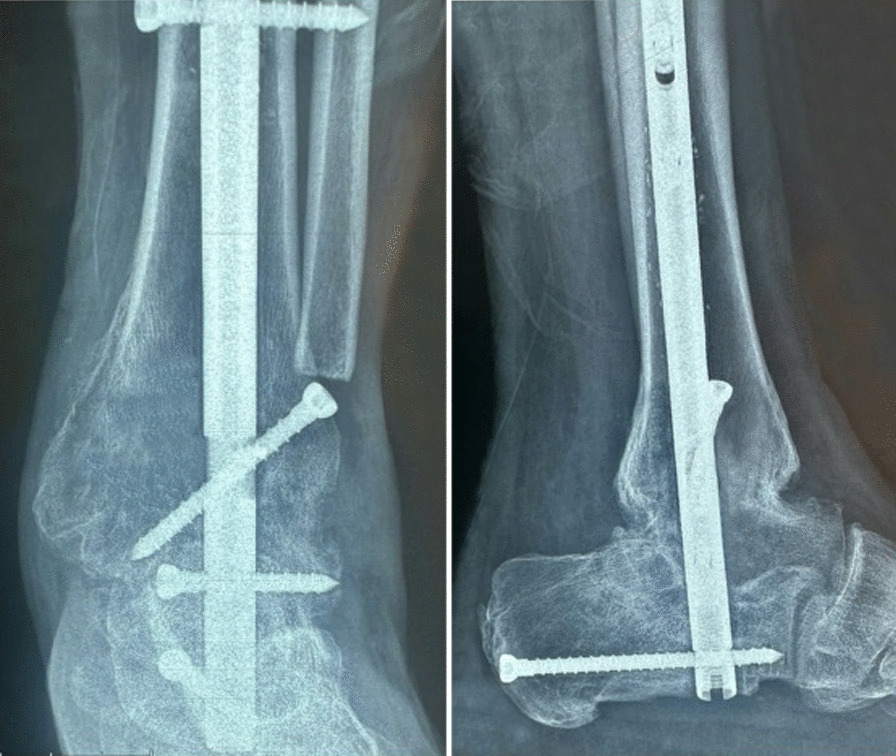
Ankle fusion with tibiotalocalcaneal nailing

The data were analyzed using SPSS 20. Descriptive statistics were initially presented for the data. Categorical variables are expressed as counts and percentages, while continuous variables are expressed as the means and standard deviations. The normality of the data was assessed using the Kolmogorov‒Smirnov test. Due to the abnormal distribution of the data, the Wilcoxon test was used for pairwise comparisons, and the Friedman test was used for dependent time comparisons with more than two groups. Median and mean rank values were used to report the figures and numbers in all tables. *P* < 0.05 was statistically significant (Fig. 1).

## Results

Overall, 26 participants were included in the study. Among the participants, 16 people (61.54%) were male. The average age of the participants was 63 ± 0.23 years, and the mean body mass index (BMI) was 27.80 ± 1.20 kg/m^2^. Smoking was reported by 19.23% [5] of the patients. 57.69% [15] of the patients had an affected right foot, and 42.31% [11] had an affected left foot. Additionally, 3.85% [1] of the patients experienced infection and nonunion after the operation. For the management of the infection, the nail was removed, and after Irrigation & Debridement, an external fixator was used (Table 1).

**Table 1:** Demographic and basic data of patients

Variables	Number (%)
Sex	
Male	16(61.54%)
Female	10(38.46%)
Smoking	
Yes	5 (19.23%)
Involved foot	
Right	15 (57.69%)
Left	11(42.31%)
Infection and nonunion after the operation	1(3.85%)
Age,(years)	63 ± 0.23
BMI, (kg/m^2^)	27.80 ± 1.20

Table 2 presents the results of the AOFAS questionnaire. The statistical analysis revealed significant differences (*P* < 0.05) between the different time points (before, after 2 weeks, and after 1 year) for several AOFAS questionnaire items. It should be noted that the patients were visited within a period of 3 months after the procedure; however, their functional status was recorded after one year.

**Table 2:** AOFAS questionnaire results of participants before, after 2 weeks and after 1 year

AOFAS Median (mean rank)	Before	After 2 weeks	After 1 year	*P* value* (before &2 week)	*P* value* (before &1year)	*P* value* (2 week &1year)	*P* value between 3 time-points **
Pain	30(1.88)	20(1.13)	40(2.98)	< 0.001	< 0.001	< 0.001	< 0.001
Move	4(1.58)	4(1.46)	7(2.96)	0.157	< 0.001	< 0.001	< 0.001
The length of the walk	4(2.38)	0(1.04)	4(2.58)	< 0.001	< 0.001	< 0.001	< 0.001
Walking surfaces	0(1.67)	0(1.42)	3(2.90)	0.025	< 0.001	< 0.001	< 0.001
Walking disorder	4(1.90)	0(1.13)	8(2.96)	< 0.001	< 0.001	< 0.001	< 0.001
Sagittal	2(2.50)	0(1.75)	0(1.75)	< 0.001	< 0.001	0.999	< 0.001
Back leg	0(2.31)	0(1.85)	0(1.85)	0.005	< 0.001	0.999	< 0.001
sustainability	0(1.02)	8(2.52)	8(2.46)	< 0.001	< 0.001	0.217	< 0.001
Alignment	5(1.02)	10(2.52)	10(2.46)	< 0.001	< 0.001	0.317	< 0.001
Total Score	49(1.81)	42(1.19)	80 (3)	0.117	< 0.001	< 0.001	< 0.001

*Wilcoxon test

**Friedman test

Table 2 shows that there were significant differences in all items before and after 1 year (*P* < 0.001). The median total score significantly improved from 49 (mean rank 1.81) at baseline to 80 (mean rank 3) after 1 year (*P* < 0.001).

Table 3 presents the results of the EQ5D5L questionnaire, which assesses health-related quality of life. The statistical analysis revealed a significant improvement (*P* < 0.05) in the total score between the different time points. The median total score significantly worsened from 65 (mean rank 1.88) at baseline to 57 (mean rank 1.23) after 2 weeks and further improved to 80 (mean rank 2.79) after 1 year (*P* = 0.002).

**Table 3:** EQ5D5L questionnaire results of participants before, after 2 weeks and after 1 year

EQ5D5L Median (mean rank)	Before	After 2 weeks	After 1 years	*P* value * (before &2 week)	*P* value * (before &1year)	*P* value* (2 week &1year)	*P* value between 3 time-points **
Total Score	65(1.88)	57(1.23)	80(2.79)	< 0.001	0.002	< 0.001	< 0.001

*Wilcoxon test

**Friedman test

## Discussion

The older theory of cause of neuropathic arthropathy is loss of sensation combined with autonomic nerve dysfunction. A newer theory is that Charcot foot is caused by loss of protective sensation and motor neuropathy causing tendon imbalance which increases forces on the foot causing deformity, subluxation, fractures and ulceration [4, 13–15]. Rosebloom found about 90% of patients with diabetes have peripheral neuropathy [16]. But only 8.5% of diabetics will develop Charcot foot [17, 18].

Surgical intervention was usually reserved for managing deformity and ulceration during the chronic phase of Charcot foot [19]. The more recent approach of early tendon balancing ± percutaneous removal of plantar bone prominence with a small bur (exostectomy) in an early phase can prevent progression of deformity, ulceration and amputation without more risky and extensive procedures [4].

Several authors have reported positive clinical outcomes with arthrodesis in Charcot neuroarthropathy patients. However, determining the most suitable device for this procedure remains a challenge [20].

The management of diabetic neuroosteoarthropathy poses significant challenges for the orthopedic community and remains controversy [1]. Specifically, when dealing with nonplantigrade alignment of the midfoot and hindfoot, there is a high incidence of skin damage and ulcers at the site of bony deformities [1, 21]. Pinzur and other authors have outlined the primary goals in treating Charcot's feet, which include achieving a foot that is free from infection and ulcers in the long term, enabling the use of commercially available depth-inlay shoes and custom-accommodative foot orthoses, and maintaining long-term walking independence [22, 23].

Reconstruction arthrodesis techniques for the treatment of Charcot's feet vary, ranging from external fixation methods using ring fixators to internal techniques involving intra- and extramedullary implants such as plates, screws, or bolts or a combination of these approaches [14, 15, 24–26]. Postoperatively, extended healing periods may be required due to comorbidities associated with diabetes, such as peripheral artery disease, which can lead to complications such as infections, nonunion or malunion, stress fractures, fixation failure, metal-induced soft-tissue irritations, implant breakage, or loosening. Consequently, the reoperation rates associated with these complications are high [7]. Currently, there is a lack of general evidence-based treatment algorithms, and the literature provides inconsistent recommendations regarding the ideal treatment type and timing [7].

Our study showed significant improvements in the AOFAS score, which includes pain score, length of the walk, walking surface, walking disorder, sagittal alignment, back leg alignment, sustainability, alignment, and the total score. The EQ5D5L questionnaire also showed a significant improvement in the total score. These findings suggest that tibiotalocalcaneal arthrodesis with hindfoot nailing can lead to positive outcomes in diabetic patients with Charcot foot joints.

Eschler et al. conducted a study on arthrodesis of the medial column and reported that approximately 50% of patients expressed satisfaction with the treatment and experienced pain relief. In our study, we observed similar outcomes, with patient satisfaction and pain reduction. However, Eschler et al. also reported a high rate of minor and major complications, with only 2 out of 21 patients experiencing complication-free healing [27]. In contrast, our study demonstrated a significantly lower rate of complications, with less than 5% of patients experiencing complications.

Jin-Soo et al. employed a dorsal-modified sliding calcaneal plate for midfoot arthrodesis and achieved successful bone union in all 10 patients within 4 months. The satisfaction rate in their study was in line with that of other procedures. In our study, we also observed successful bone union, with a similar time frame for healing [23]. However, we did not need for a second surgery, which was required in 20% of patients in Jin-Soo et al.'s study.

Dalla Paola et al. enrolled 18 diabetic patients with hindfoot Charcot neuroarthropathy and reported limb salvage in all patients. Fourteen patients achieved complete bony union of ankle arthrodesis [28]. These results align with our study, which also demonstrated successful limb salvage and favorable outcomes in ankle arthrodesis, with a high percentage of patients achieving complete bony union. Lee et al. obtained similar results in their study involving seven patients [29].

Caravaggi et al. studied a cohort of 45 diabetic patients with Charcot neuroarthropathic ankle deformity and suggested performing ankle and hindfoot arthrodesis with an intramedullary nail during the early chronic stage of the disease. They suggested that this approach may reduce the risk of progressive deformation and complications [30]. Our study supports these findings, as we also recommend early surgical reconstruction to minimize the risk of complications in a similar patient population.

Yammine et al. conducted a comprehensive meta-analysis comparing external fixation and intramedullary nailing in Charcot neuroarthropathy patients. They found that the external fixation group had a greater rate of hardware and wound infection than did the intramedullary nailing group. The fusion rate was also greater in the intramedullary nailing group, while the amputation rate was lower [31]. These results align with our study, which also demonstrated a low rate of complications and a high fusion rate in patients treated with intramedullary nailing.

Our study has several limitations. First, the study was carried out between January 2020 and December 2021, restricting the ability to assess long-term outcomes beyond the 1-year follow-up period. Moreover, the data collection relied on self-reported measures and subjective assessments, which may introduce measurement bias. Furthermore, the study lacked a control group, making it difficult to assess the effectiveness of the treatment compared to alternatives. The improvement in outcomes (i,e quality of life, pain) could result from placebo (i,e individual bias) due to the absence of a double-blind procedure and future double-blinded RCT is needed. Also, further studies should include a longer follow-up period.

## Conclusion

Overall, the present study provides further evidence supporting the effectiveness of tibiotalocalcaneal arthrodesis by hindfoot nailing in diabetic patients with Charcot deformity of ankle ± subtalar joints. The study demonstrated comparable or superior outcomes in terms of patient satisfaction, pain reduction, complication rate, bone union, limb salvage, and fusion rate when compared to previous studies.

## Data Availability

The data that support the findings of this study are available from the corresponding author upon reasonable request.
